# Diagnostics, Management, and Outcomes in Patients with Pyogenic Spinal Intra- or Epidural Abscess

**DOI:** 10.3390/jcm12247691

**Published:** 2023-12-14

**Authors:** Mido Max Hijazi, Timo Siepmann, Ibrahim El-Battrawy, Assem Aweimer, Kay Engellandt, Dino Podlesek, Gabriele Schackert, Tareq A. Juratli, Ilker Y. Eyüpoglu, Andreas Filis

**Affiliations:** 1 Department of Neurosurgery, Division of Spine Surgery, Faculty of Medicine, Technische Universität Dresden, University Hospital Carl Gustav Carus, Fetscherstrasse 74, 01307 Dresden, Germany; dino.podlesek@ukdd.de (D.P.); gabriele.schackert@ukdd.de (G.S.); tareq.juratli@ukdd.de (T.A.J.); ilker.eyuepoglu@ukdd.de (I.Y.E.); afilis@neuromaster.gr (A.F.); 2 Department of Neurology, Faculty of Medicine, Technische Universität Dresden, University Hospital Carl Gustav Carus, Fetscherstrasse 74, 01307 Dresden, Germany; timo.siepmann@ukdd.de; 3 Department of Cardiology, Bergmannsheil University Hospital, Ruhr University Bochum, Bürkle De La Camp-Platz 1, 44789 Bochum, Germany; ibrahim.elbattrawy2006@gmail.com (I.E.-B.); assem.aweimer@bergmannsheil.de (A.A.); 4 Institute of Diagnostic and Interventional Neuroradiology, Faculty of Medicine, Technische Universität Dresden, University Hospital Carl Gustav Carus, Fetscherstrasse 74, 01307 Dresden, Germany; kay.engellandt@ukdd.de

**Keywords:** spinal intradural abscess, spinal epidural abscess, epidural empyema, *Staphylococcus aureus*, spinal infection

## Abstract

Background: Owing to the lack of evidence on the diagnostics, clinical course, treatment, and outcomes of patients with extremely rare spinal intradural abscess (SIA) and spinal epidural abscess (SEA), we retrospectively analyzed and compared a cohort of patients to determine the phenotyping of both entities. Methods: Over a period of 20 years, we retrospectively analyzed the electronic medical records of 78 patients with SIA and SEA. Results: The patients with SIA showed worse motor scores (MS scores) on admission (SIA: 20 ± 26 vs. SEA: 75 ± 34, *p* < 0.001), more often with an ataxic gait (SIA: 100% vs. SEA: 31.8%, *p* < 0.001), and more frequent bladder or bowel dysfunction (SIA: 91.7% vs. SEA: 27.3%, *p* < 0.001) compared to the SEA patients. Intraoperative specimens showed a higher diagnostic sensitivity in the SEA patients than the SIA patients (SIA: 66.7% vs. SEA: 95.2%, *p* = 0.024), but various pathogens such as *Staphylococcus aureus* (SIA 33.3% vs. SEA: 69.4%) and *Streptococci* and *Enterococci* (SIA 33.3% vs. SEA: 8.1%, *p* = 0.038) were detected in both entities. The patients with SIA developed sepsis more often (SIA: 75.0% vs. SEA: 18.2%, *p* < 0.001), septic embolism (SIA: 33.3% vs. SEA: 8.3%, *p* = 0.043), signs of meningism (SIA: 100% vs. 18.5%, *p* < 0.001), ventriculitis or cerebral abscesses (SIA: 41.7% vs. SEA: 3.0%, *p* < 0.001), and pneumonia (SIA: 58.3% vs. SEA: 13.6%, *p* = 0.002). The mean MS score improved in both patient groups after surgery (SIA: 20 to 35 vs. SEA: 75 to 90); however, the SIA patients showed a poorer MS score at discharge (SIA: 35 ± 44 vs. SEA: 90 ± 20, *p* < 0.001). C-reactive protein (CrP) (SIA: 159 to 49 vs. SEA: 189 to 27) and leukocyte count (SIA: 15 to 9 vs. SEA: 14 to 7) were reduced at discharge. The SIA patients had higher rates of disease-related mortality (SIA: 33.3% vs. SEA: 1.5%, *p* = 0.002), had more pleural empyema (SIA: 58.3% vs. SEA: 13.6%, *p* = 0.002), required more than one surgery (SIA: 33.3% vs. SEA 13.6%, *p* = 0.009), were treated longer with intravenous antibiotics (7 ± 4 w vs. 3 ± 2 w, *p* < 0.001) and antibiotics overall (12 ± 10 w vs. 7 ± 3 w, *p* = 0.022), and spent more time in the hospital (SIA: 58 ± 36 vs. SEA: 26 ± 20, *p* < 0.001) and in the intensive care unit (SIA: 14 ± 18 vs. SEA: 4 ± 8, *p* = 0.002). Conclusions: Our study highlighted distinct clinical phenotypes and outcomes between both entities, with SIA patients displaying a markedly less favorable disease course in terms of complications and outcomes.

## 1. Introduction

Spinal intradural abscess (SIA) is an extremely rare form of primary spinal infection (PSI) [1]. Together with isolated spinal epidural abscess (SEA) and the most common form, spondylodiscitis (SD), they form PSI [2,3]. While the incidence of PSI was previously reported to be 0.2–3 cases per 100,000 people per year [4,5,6], the current age-standardized incidence rate in Germany is estimated to be 30 per 250,000 people a year based on data from the Federal Statistical Office (2015) [7].

SIAs are located in the subdural space or in the spinal cord. In contrast to spinal epidural abscesses, intradural abscesses can occur anywhere along the spinal cord or in the spinal cord and are associated with a higher mortality rate. In the first instance, SIA and isolated SEA are due to the hematogenous spread of an infection, but they can also be caused iatrogenically, e.g., by epidural injections [3,8].

In the case of neurological deficits in SIA or SEA patients, the standard recommended treatment is surgical drainage followed by adequate antimicrobial therapy for 4–6 weeks [2,9,10,11,12].

The prognosis of an isolated SEA may be better than that of SD with or without an epidural abscess [13]. If SIA is not treated immediately, the prognosis is poor [14,15]. The incidence, clinical course, morbidity, mortality, and optimal standardized treatment of patients with SIA are still unclear, and most evidence is based on case reports and lacks systematic analysis [2,10].

Given the lack of conclusive evidence on the diagnosis, clinical course, treatment, and outcome of patients with SIA and SEA, we retrospectively analyzed a large cohort of 78 patients and compared both populations to determine the phenotyping of the two entities and propose a treatment algorithm for both diseases.

## 2. Materials and Methods

### 2.1. Study Design and Patient Data

#### 2.1.1. Study Design

We retrospectively analyzed the data of 228 patients with PSI. We included all consecutive patients with PSI who underwent surgery at our hospital between 2002 and 2022. Patients were excluded based on one of the following criteria:

- Early or late postoperative local spinal infection
- Only conservative treatment
- Spondylodiscitis

**Figure 1:**
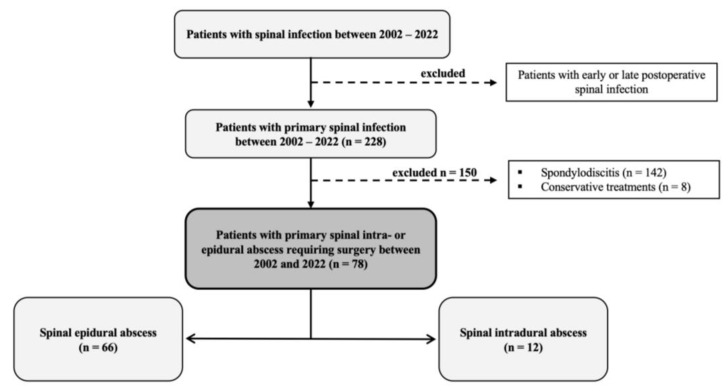
Study design. This figure shows our study design in 228 patients with primary spinal infection. One-hundred and fifty patients were excluded due to spondylodiscitis and only receiving conservative treatment.

We investigated 78 consecutive patients with SIA and SEA who underwent surgery at our neurosurgical university spine center in Dresden, Germany, between 2002 and 2022 (Figure 1).

#### 2.1.2. Institutional Review Board and Electronic Patient Data Software

This study was reviewed and approved by the local ethics committee of the Medical Faculty of the TU Dresden and University Hospital Carl Gustav, Dresden (Ref: BO-EK-17012022). Data on the patients were identified using the ORBIS system (ORBIS, Dedalus, Bonn, Germany), and imaging files were identified using the IMPAX system (IMPAX, Impax Asset Management Group plc, London, UK).

#### 2.1.3. Patient Data

The electronic medical records were pseudonymized and first divided into two groups—SIA or SEA—according to the type of infection. The data collected included sex, age, type of PSI, causative pathogen, detection via (blood cultures, intraoperative specimens, and computer tomography (CT)-guided biopsies), time passed to pathogen detection, type of antibiotic strategy, presence of psoas or pleural abscesses, spinal location of infection, time passed to surgery, incidental dural tears, primary source of infection, surgical and antibiotic treatment, type of surgical procedure, comorbidities (diabetes mellitus, immunodeficiency, obesity, malignancy, hepatic cirrhosis, dialysis, stent or vascular prosthesis, artificial heart valve replacement, osteoporosis, rheumatoid arthritis or elevated rheumatoid factors, gout or elevated uric acid, chronic venous insufficiency, peripheral artery disease, and atrial fibrillation), relapse, sepsis, septic embolism, sign of meningism, reoperation, disease-related mortality, hospitalization, intensive care unit (ICU) stay, ventriculitis or cerebral abscess, deep wound infection, pneumonia, urinary tract infection, presentation of ataxic gait, bladder or bowel dysfunction, number of decompressed levels, number of required surgeries, American Society of Anaesthesiologists (ASA) classification, preoperative and postoperative C-reactive protein (CrP) level and leukocyte count, and motor score based on the American Spinal Injury Association grading system (MS score).

### 2.2. Clinical Management

We diagnosed SIA and SEA on the basis of the patient’s medical history, clinical examination, fever, laboratory values such as leukocyte count, CrP and procalcitonin, typical radiological changes in magnetic resonance imaging (MRI) and CT, as well as pathogen detection in blood cultures, intraoperative specimens, or CT-guided biopsies of paravertebral psoas abscesses. The determination of whether a patient had an SIA or an SEA was based on the intraoperative findings in accordance with the MRI.

Depending on the clinical condition, at least two blood cultures were taken before antibiotic therapy was started for microbiological diagnosis, although some of the patients were initially treated with antibiotics in a peripheral hospital due to their severe clinical condition.

All the patients underwent transthoracic echocardiography (TTE) to rule out infective endocarditis (IE), while the patients with possible or definite IE according to the modified Duke criteria or with proven Gram-positive bacteria received transesophageal echocardiography (TEE).

The SEA and SIA cases were discussed in our neurosurgery–neuroradiology conference or multidisciplinary spine board and treated in collaboration with infectiologists, neuroradiologists, trauma surgeons, and orthopedic surgeons to determine the best treatment strategy for the patients.

### 2.3. Surgical and Antibiotic Treatment

Surgical treatment of SEA was indicated in the case of source control, epidural abscess with space-occupying effects, or neurological deficits. All the patients with SIA had severe motor deficits and elevated infection parameters and showed obvious intradural findings on their post-contrast MRI. The type of surgical intervention was determined in the multidisciplinary spine board or in the neurosurgical–neuroradiological conference.

The patients with SEA underwent abscess evacuation with epidural suction–irrigation drainage (ESID) or anterior cervical discectomy and fusion (ACDF) for abscesses ventral to the cervical spinal cord with ESID. The patients with SIA were treated via a laminectomy or hemilaminectomy, midline durotomy, and abscess evacuation if the abscess was subdural and extramedullary. In the case of an intramedullary abscess, a limited midline myelotomy with evacuation of the abscess was performed. However, to avoid postoperative cerebrospinal fluid (CSF) leakage, we decided not to use ESID in SIA. Therefore, surgical decision making depended on clinical experience and various defined radiological features.

All the patients received targeted antibiotic treatment (TAT) or empirical antibiotic treatment (EAT) depending on their clinical condition upon admission and according to the recommendations of the local infectious diseases department. It is important to mention that most of the patients with EAT came to us from peripheral hospitals and needed immediate surgery; therefore, the number of EAT patients was high. EAT was switched to TAT as soon as the causative pathogens were isolated.

In the case of a culture-negative spinal infection (blood culture, image-guided biopsy, and open surgery), we initiated EAT. EAT was based on the suspected pathogen, suspected source of infection, clinical condition, epidemiologic risk, and local historical in vitro susceptibility data. In most cases, our therapy consisted of a combination of vancomycin with ceftriaxone or vancomycin with piperacillin/tazobactam.

The first-line therapy in our department for methicillin-sensitive *Staphylococcus aureus* (MSSA) was flucloxacillin (1.5–2 g intravenously (IV) every (e) 4–6 h), β-hemolytic *Streptococci* or penicillin-susceptible *Enterococci* penicillin G (20–24 million units IV e 24 h), *Enterobacteriaceae* or *Pseudomonas aeruginosa* Cefepime (2 g IV e 8–12 h), methicillin-resistant *Staphylococcus aureus* (MRSA), or coagulase-negative *Staphylococci* (CoNS) or penicillin-resistant *Enterococci* Vancomycin (IV 15–20 mg/kg e 12 h with a loading dose and monitoring of serum levels). In the patients with foreign body infections or osteosynthesis material, a combination such as flucloxacillin or vancomycin with rifampicin was used [7,16].

Depending on the clinical condition, infection parameters, and MRI findings, the SIA patients were switched from IV to oral antibiotics after 4 weeks for a further 6–8 weeks. The twelve SIA patients in our study were treated longer on average (7 weeks) with IV antibiotics due to their severe infection after consultation with our infectiologist. The SEA patients were switched from IV antibiotics to oral antibiotics after 2–3 weeks on average for a further 4 weeks.

All the patients who met our recommendation were followed up clinically and radiologically at 3, 6, and 12 months after discharge from the hospital, if possible.

### 2.4. Illustrative Case with Spinal Intradural Abscess

**Figure 2:**
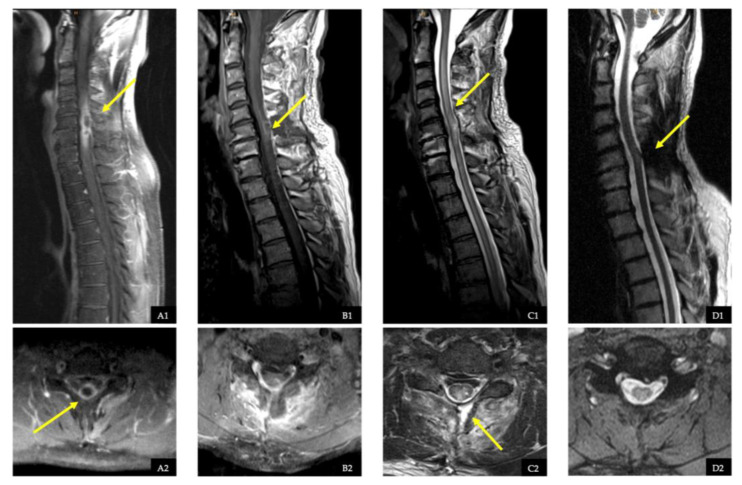
Clinical management of spinal intradural abscess. (**A1**,**A2**): Preoperative sagittal and axial contrast-enhanced T1-weighted MR images of the cervical spine showing a spinal intradural abscess (SIA) on the dorsal part of the spinal cord at the level of C5 to C7. The yellow arrows indicate the SIA. (**B1**,**B2**): Immediate postoperative sagittal and axial T1-weighted contrast-enhanced MR images of the cervical spine showing postoperative contrast enhancement at the level of C6 (yellow arrow). (**C1**,**C2**): Immediate postoperative sagittal and axial T2-weighted MR images showing the postoperative intramedullary hyperintense signal (sign of myelopathy, yellow arrow in C1). The yellow arrow in C2 points out the approach. (**D1**,**D2**): Sagittal and axial T2-weighted MR images after 12 weeks at the time of discontinuation of antibiotics, showing no signs of infection.

A 49-year-old male patient presented with a three-day history of an increasing ataxic gait, high-grade tetraparesis (MS score 55), urinary and faecal incontinence, meningism sign, and positive Babinski sign. Infectious parameters, including leukocyte count and CrP, were elevated, and MRI scans of the whole spine and cranium with contrast showed an isolated dorsal SIA between the C5 and C7 levels. Intramedullary involvement could not be ruled out. Emergency surgery was performed in the form of a dorsal laminectomy at the C6 level, durotomy, and abscess evacuation, as well as irrigation with gentamycin. Intramedullary involvement was excluded intraoperatively via ultrasound. The postoperative MRI with contrast showed a regression of the SIA, but with residual edema of the spinal cord. *Streptococcus anginosus* was detected, which was treated with antibiotics for a total of 12 weeks (4 weeks intravenously). A source of infection could not be determined through further examinations. Endocarditis and septic embolisms were ruled out. On discharge, the infection parameters were within the normal range, and the tetraparesis had regressed (MS score 85). At the first outpatient follow-up after completion of antibiotic therapy (12 weeks), the patient had no neurological abnormalities, and no recurrence was detected on the postcontrast MRI (Figure 2).

### 2.5. Illustrative Case with Spinal Epidural Abscess

**Figure 3:**
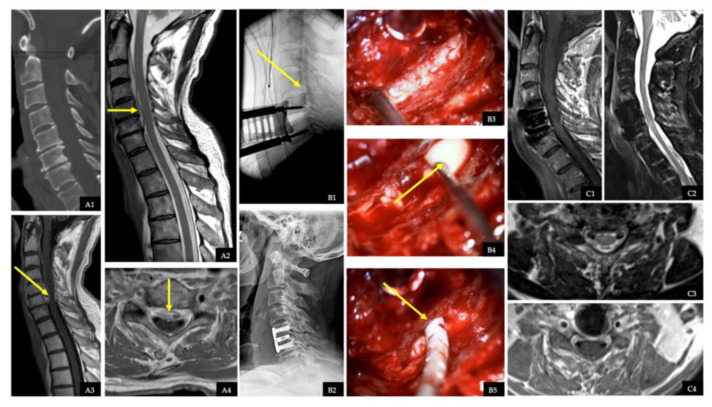
Clinical management of spinal epidural abscess. (**A1**): Preoperative sagittal reformatted CT image showing no sign of spondylodiscitis. (**A2**): Preoperative sagittal T2-weighted MR image of the cervical spine showing an isolated spinal epidural abscess (SEA) on the ventral part of the spinal canal at the level of C2 to C7 (yellow arrow). (**A3**,**A4**): Preoperative sagittal and axial contrast-enhanced T1-weighted MR images presenting the SEA (yellow arrows). (**C3**,**C4**): Preoperative sagittal and axial contrast-enhanced T1-weighted MR images of the cervical spine showing an SEA (yellow arrows). (**B1**): Intraoperative sagittal X-ray showing the insertion of epidural drainage from the C5/C6 level to C2 (yellow arrow). (**B2**): Postoperative sagittal X-ray image showing the anterior cervical discectomy and fusion (ACDF). (**B3**): Intraoperative image presenting the situation after discectomy of C5/C6 without signs of infection. (**B4**): Intraoperative image showing the pus outflow from the posterior longitudinal ligament after opening it (yellow arrow). (**B5**): Intraoperative image revealing the insertion of the catheter up to the level of C2 (yellow arrow). (**C1**–**C4**) Sagittal and axial T2-weighted and contrast-enhanced T1-weighted MR images at the time of discontinuation of antibiotics showing no signs of infection.

A 68-year-old male patient presented to a peripheral hospital with one week of increasing neck pain, signs of meningism, and fever. The infection parameters, including leukocyte count and CrP, were elevated, and *Streptococcus dysgalactiae* was detected in his blood cultures. The patient was then treated with intravenous antibiotics. A postcontrast MRI of the entire spine and cranium revealed a space-occupying isolated SEA at the level of C2 to C7 ventral to the spinal cord. We performed urgent surgery in form of an ACDF at level C5/6, abscess evacuation, and irrigation with Gentamycin through an epidural irrigation catheter up to level C2. The intraoperative specimen also contained *Streptococcus dysgalactiae*. Intravenous antibiotics were administered for 2 weeks and then continued orally for 4 weeks. A source of infection could not be determined through further examinations. Endocarditis and septic embolisms were ruled out. On discharge, the infection parameters were within the normal range and there were no neurological abnormalities. No recurrence was detected during the MRI examination with contrast (Figure 3).

### 2.6. Statistical Analysis

Statistical analysis of the data was performed using the SPSS software package (SPSS Statistics 29, IBM, Armonk, NY, USA). Descriptive statistics were used, and the categorical variables were tested using Fisher exact tests or chi-square tests. The numerical variables were analyzed using Mann–Whitney U tests. All the statistical tests were two-sided, and a *p* value (*p* < 0.05) was considered statistically significant.

## 3. Results

### 3.1. Baseline Characteristics

Over a 20-year period, 66 patients were diagnosed with SEA and 12 patients were diagnosed with SIA. The groups did not differ in terms of age, gender, preoperative infection parameters, and spinal localization of the infection.

On admission, a new motor deficit was observed in both groups, with the mean MS score being lower in SIA than in SEA (SIA: 20 ± 26 vs. SEA: 75 ± 34, *p* < 0.001). The patients with SIA had a more ataxic gait (SIA: 12, 100% vs. SEA: 21, 31.8%, *p* < 0.001) and bladder or bowel dysfunction (SIA: 11, 91.7% vs. SEA: 18, 27.3%, *p* < 0.001) compared to the SEA patients.

The most common comorbidities showed only a difference in the distribution of rheumatic diseases (SIA: 0, 0.0% vs. SEA: 9/25, 36.0%, *p* = 0.018) and gout (SIA: 0, 0.0% vs. SEA: 9/21, 42.9%, *p* = 0.012) between the two groups.

The American Society of Anesthesiologists (ASA) class was significantly higher in the SIA patients, such as ASA class IV (SIA: 4, 33.3% vs. SEA: 4, 6.1%, *p* = 0.028), and the distribution of the source of infection, such as skin infection (SIA: 1/6, 16.7% vs. SEA: 14/44, 31.8%, *p* = 0.015) (Table 1).

### 3.2. Pathogens and Diagnostic Sensetivity

The distribution of pathogens between the two groups was significantly different, such as for MSSA (SIA: 3/9, 33.3% vs. SEA: 43, 69.4%, *p* = 0.038) and *Streptococci* and *Enterococci* (SIA: 3/9, 33.3% vs. SEA: 5, 8.1%, *p* = 0.038).

In SEA, pathogens could always be detected, while no pathogens could be detected in 25.0% of the SIA patients (n = 3). No polymicrobial infections were identified in the SEA patients, while this was the case in 33.3% of the SIA patients (n = 3, *p* = 0.003).

The diagnostic sensitivity of the intraoperative specimens was higher in both groups than that of the blood culture and CT-guided biopsy. The success rate via intraoperative specimens was significantly higher in SEA than in SIA (SIA: 6/9, 66.7% vs. SEA: 59, 95.2%, *p* = 0.024) (Table 2).

### 3.3. Antibiotic Management and Surgical Characteristics

The patients with SIA were treated longer with intravenous antibiotics (SIA: 7 ± 4 w vs. SEA: 3 ± 2 w, *p* < 0.001), and they also received prolonged antibiotics overall (SIA: 12 ± 10 w vs. SEA: 7 ± 3 w, *p* = 0.022) compared to the SEA patients. The mean postoperative MS score was lower in the SIA patients than in the SEA patients (SIA: 35 ± 44 vs. SEA: 90 ± 20, *p* < 0.001).

Pleural empyema was observed more frequently in the SIA patients than in the SEA patients (SIA: 7, 58.3% vs. n SEA: 9, 13.6%, *p* = 0.002). The patients with SIA were more likely to require more than one surgery compared to the SEA patients (SIA: 4, 33.3% vs. SEA 9, 13.6%, *p* = 0.009) (Table 3).

### 3.4. Disease Course and Complications

The patients with SIA developed more sepsis (SIA: 9, 75.0% vs. SEA: 12, 18.2%, *p* < 0.001), septic embolism (SIA: 4, 33.3% vs. SEA: 4/48, 8.3%, *p* = 0.043), signs of meningism (SIA: 12, 100% vs. 12/65, 18.5%, *p* < 0. 001), ventriculitis or cerebral abscess (SIA: 5, 41.7% vs. SEA: 2, 3.0%, *p* < 0.001), pneumonia (SIA: 7, 58.3% vs. SEA: 9, 13.6%, *p* = 0.002), and disease-related mortality (SIA: 4, 33.3% vs. SEA: 1, 1.5%, *p* = 0.002).

Furthermore, the patients with SIA spent more time in the hospital (SIA: 58 ± 36 vs. SEA: 26 ± 20, *p* < 0.001) and in the intensive care unit (SIA: 14 ± 18 vs. SEA: 4 ± 8, *p* = 0.002) (Table 4).

### 3.5. Course of the Disease within a Group

**Figure 4:**
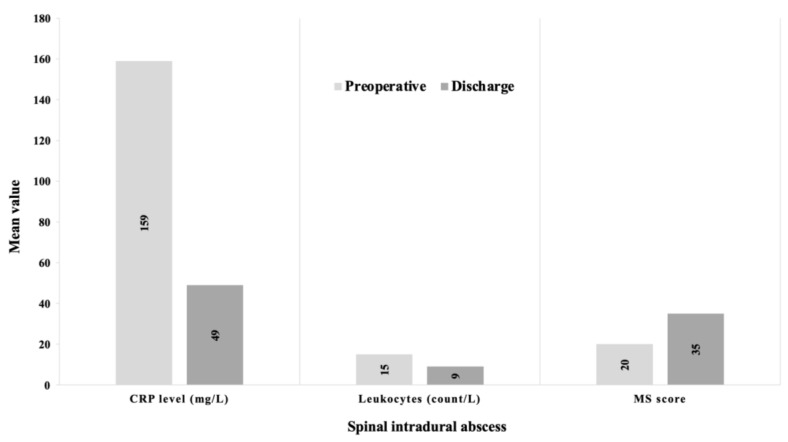
Clinical course of spinal intradural abscess. This figure shows the course of CrP (C-reactive protein), leukocyte count, and the MS score for motor deficits (motor score of the American Spinal Injury Association Grading System) in spinal intradural abscess patients.

**Figure 5:**
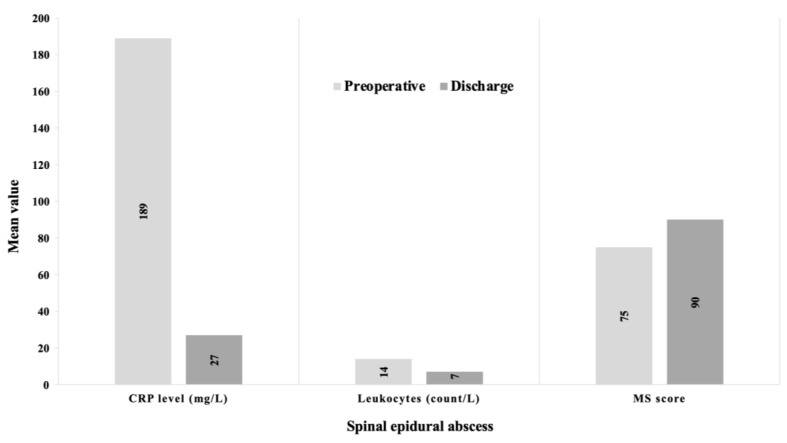
Clinical course of spinal epidural abscesses. This figure shows the course of CrP (C-reactive protein), leukocyte count, and the MS score for motor deficits (motor score of the American Spinal Injury Association Grading System) in spinal epidural abscess patients.

The mean motor deficits based on the MS score improved in both groups (SIA: from 20 to 35 vs. SEA: from 75 to 90) following surgery. In addition, infection parameters such as CrP (SIA: from 159 to 49 vs. SEA: from 189 to 27) and leukocyte count (SIA: from 15 to 9 vs. SEA: from 14 to 7) were reduced at discharge (Figure 4 and Figure 5).

## 4. Discussion

The main message of this study and our 20-year experience with SIA and SEA patients is that SIA is a very severe and rare entity with a distinctly less-favorable disease course in terms of complications and outcomes, requiring a long, intensive conservative and surgical treatment.

The baseline demographics of the patients in our study did not differ between the two groups in terms of age, gender, preoperative infection parameters, and spinal localization of the infection. To the best of our knowledge, there are no comparisons in the literature between the two groups, as SIA being a rare disease [2,13,17,18]. A systematic review by Arko et al. showed an average age of 57.2 years in 1099 patients with SEA, which was similar to our SEA patients, with an average age of 59 years [19]. Likewise, a study by Lenga et al. found an average age of 69.6 years in patients with SIA, which is also comparable to our average age of SIA patients of 63 years [2]. Presumably, SEA and SIA are more common in men than in women, which we could not be reproduced in our study [3,19]. In our study, the patients with SEA mostly presented with infections of the lumbar spine, which has also been described in the literature [19]. On the contrary, infection in the thoracic spine was more frequent in the SIA patients in our cohort.

The symptoms on admission, such as new motor deficits based on the MS score, an ataxic gait, and bladder or bowel dysfunction, were worse in the SIA patients compared with the SEA patients in our study, although both groups showed significant improvement at discharge after medical and surgical treatment [2,18,20,21]. This study demonstrates the severity of SIA and the importance of surgical and medical treatment for SIA patients.

Remarkably, the SEA patients had more rheumatic diseases and gout as a concomitant disease. The extent to which these two diseases have an influence on the development of SEA needs to be investigated in a prospective study. Intravenous drug abuse is suggested to be an associated risk factor, and diabetes is the most commonly associated medical comorbidity in SEA patients [19]. In our study, we found that obesity (BMI > 30 kg/m²), diabetes mellitus, osteoporosis, rheumatic diseases, and gout were frequent in the SEA patients, whereas malignancy, liver cirrhosis, diabetes, and obesity were common in the SIA patients. The poorer ASA class in the SIA patients compared to the SEA patients also reflected the severity of the diseases and sequelae.

The primary source of infection in our cohort was different for both entities, with the most common reason being due to epidural intervention followed by skin infection. These two causes have also been identified in the literature as the most common primary sources of infection [3,13,17,18,20,22]. The finding of the primary source of infection is essential in the management of both diseases. We were only able to detect the primary nidus in 50% of the patients with SIA, whereas we identified the primary infectious source in 82% of the SEA patients.

In order to successfully treat a bacterial infection, pathogen isolation is absolutely crucial. In our SEA patients, as known in the literature, MSSA was the most common pathogen, followed by *Streptococci* and *Enterococci* and coagulase-negative *Staphylococci* [13,19]. The patients with SIA in our cohort showed a diverse distribution of pathogens, such that 33% of the patients had polymicrobial infections, 33% had MSSA, and 33% had *Streptococci* and *Enterococci*. Lenga et al. pointed out that MSSA is the most common pathogen in SIA [2].

Open surgery specimens showed the best diagnostic sensitivity for both entities, with SIA patients having a poorer sensitivity. Nevertheless, the collection of blood cultures plays a greater role in pathogen isolation due to its natural non-invasiveness [23].

In our cohort, most of the patients with both entities were treated with the EAT strategy, and it took on average 4–6 days to switch to TAT. Overall, the antibiotic treatment was effective, and both groups showed a decrease in the infection parameters (CrP and leukocyte count) at discharge. The duration of intravenous antibiotic therapy and the total duration of antibiotic therapy were significantly longer in SIA, which is consistent with our algorithm.

Pleural abscesses were more common in our SIA patients than in our SEA patients, whereas the rate of paravertebral psoas abscesses was equal in both groups, indicating the frequent thoracic localization and systematic infection of the SIA patients. On average, the patients were admitted within 2 days of admission. This slight delay is probably due to the extensive diagnostics that these patients needed. An incidental durotomy occurred intraoperatively in approximately 10% of the SEA patients in our cohort. However, the rates of incisional durotomies in lumbar spine surgeries is reported in the literature to be between 1 and 17% [24]. Most of the patients with SEA and SIA were treated equally with one or two levels of spinal decompression and abscess evacuation, with the SIA patients more often requiring more than one surgery on average.

In contrast to our expectations, the occurrence of IE in both entities seemed to be a rarity, whereas PSI patients, such as those with spondylodiscitis with MSSA as a pathogen, showed a higher occurrence of IE in the literature [13,25,26,27]. All the SIA patients underwent TTE followed by TEE without finding vegetations confirming IE, although some of these patients had septic emboli, brain abscesses, and ventriculitis with proven bacteremia. The largest study of patients with SIA provided no insights into endocarditis as a possible complication or cause in its results [2]. This finding should be investigated in a larger prospective multicenter study. SEA often results from local infection following spinal infiltration or epidural analgesia via a catheter; therefore, IE is less likely.

Both groups showed a recurrence rate of 8–25%, with no significant difference. There were no significant differences in the rates of deep wound infections, between 14–17%, in both groups. Urinary tract infections occurred more frequently in the SIA patients without being significant. The patients with SIA in our cohort developed more sepsis, septic embolism, signs of meningism, ventriculitis or cerebral abscess, and pneumonia. These complications demonstrate the severity of SIA and the difficulties of its management, which requires multidisciplinary assessments.

Disease-related mortality in SEA patients is reported in the literature to be between 1.3% and 31%; however, in our cohort, it was 1.5% [13,28]. Indeed, SIA is associated with high morbidity and mortality rates of up to 45%, which is also similar to our disease-related mortality rate of 33% in our SIA patients [29,30,31]. Furthermore, we observed in our cohort that the patients with SIA spent more time in the hospital and in the intensive care unit compared to the SEA patients.

### Limitations and Strengths of This Study

Our study is limited by its retrospective design and a possible bias in the selection of the more severe cases due to the high degree of specialization at our university center. In addition, some of the patients were primarily pre-treated in another hospital and transferred to us secondarily following a clinical deterioration. Furthermore, the generalizability of our observations is limited by the monocentric design of our study. The number of SIA patients treated was limited, with only 12 patients. Nevertheless, our data are based on detailed state-of-the-art clinical, imaging, and microbiological diagnostics with high internal validities. To validate and confirm our results, a large multicenter study with a larger number of SIA patients is needed.

## 5. Conclusions

Our 20-year experience and cohort analysis of the diagnostics and clinical management of SIA and SEA shows that both entities display different clinical phenotypes and outcomes. The patients with SIA had poorer symptoms on admission, higher ASA classes, different primary sources of infection, distinct causative pathogens, and more frequent pleural empyema.

The patients with SIA were more likely to develop sepsis, septic embolism, signs of meningism, ventriculitis or brain abscesses, and pneumonia. Intraoperative sampling has the best diagnostic sensitivity for both entities.

In addition, disease-related mortality was very high in the SIA patients, and these patients required more frequent surgeries, longer durations of intravenous antibiotic therapy, and longer overall durations of antibiotic therapy, as well as prolonged hospital stays and ICU stays.

## Figures and Tables

**Table 1:** Baseline factors between spinal intra- or epidural abscess.

Variables	SEA (n = 66)	SIA (n = 12)	*p* Value
**Demographics**			
Age, mean ± SD	63 ± 13	59 ± 21	0.739
Male, n (%)	35 (53.0)	6 (50.0)	1.000
**Preoperative symptoms**			
Preoperative MS score, mean ± SD	75 ± 34	20 ± 26	**<0.001**
Ataxic gait, n (%)	21 (31.8)	12 (100)	**<0.001**
Bladder or bowel dysfunction, n (%)	18 (27.3)	11 (91.7)	**<0.001**
**Preoperative infection parameters**			
CrP level, mg/L, mean ± SD	189 ± 111	159 ± 91	0.406
Leukocyte count/L, mean ± SD	14 + 6	15 ± 4	0.445
**ASA class, n (%)**			
I	3 (4.5)	0 (0.0)	**0.028**
II	24 (36.4)	2 (16.7)	
III	35 (53.0)	6 (50.0)	
IV	4 (6.1)	4 (33.3)	
**Medical history, n (%)**			
Obesity (BMI > 30 kg/m²)	29 (43.9)	3 (25.0)	0.340
Diabetes mellitus	18 (27.3)	3 (25.0)	1.000
Osteoporosis	19/58 (32.8)	1/11 (9.1)	0.157
Hepatic cirrhosis	7 (10.6)	3 (25.0)	0.178
Dialysis	1 (1.5)	2 (16.7)	0.060
History of malignancy	12 (18.2)	4 (33.3)	0.254
Atrial fibrillation	8 (12.1)	1 (8.3)	1.000
Rheumatic disease or elevated RF	9/25 (36.0)	0 (0.0)	**0.018**
Immunodeficiency	7 (10.6)	2 (16.7)	0.622
Gout or elevated uric acid	9/21 (42.9)	0 (0.0)	**0.012**
PAD/CVI	6 (9.1)	0 (0.0)	0.582
History of stent or vascular prosthesis	2 (3.0)	0 (0.0)	1.000
AHVR	2 (3.0)	0 (0.0)	1.000
**Localization, n (%)**			
Cervical	7 (10.6)	1 (8.3)	0.246
Cervicothoracic	5 (7.6)	2 (16.7)	
Thoracic	13 (19.7)	5 (41.7)	
Thoracolumbar	6 (9.1)	2 (16.7)	
Lumbar	25 (37.9)	1 (8.3)	
Distributed in the whole spine	10 (15.2)	1 (8.3)	
**Source of infection, n (%)**			
Skin infection	14/44 (31.8)	1/6 (16.7)	**0.015**
Foreign body-associated infection	2/44 (4.5)	0 (0.0)	
Following epidural intervention	15/44 (34.1)	2/6 (33.3)	
Respiratory tract infection	3/44 (6.8)	0 (0.0)	
Urinary tract infection	6/44 (13.6)	0 (0.0)	
Gastrointestinal tract infection	0 (0.0)	2/6 (16.7)	
Retropharyngeal infection	1/44 (2.3)	0 (0.0)	
Odontogenic	3/44 (6.8)	1/6 (16.7)	
Unknown	12/66 (18.2)	6/12 (50.0)	

SIA: spinal intradural abscess; SEA: spinal epidural abscess; MS: motor score of the American Spinal Injury Association grading system; SD: standard deviation; CrP: C-reactive protein; ASA: American Society of Anesthesiologists; BMI: body mass index, RF: rheumatoid factors; PAD: peripheral arterial disease; CVI: chronic venous insufficiency; AHVR: artificial heart valve replacement. Values in bold are significant results (*p* < 0.05), as indicated in the methods.

**Table 2:** Pathogens and diagnostic sensitivity of spinal intra- or epidural abscess.

Variables	SEA (n = 66)	SIA (n = 12)	*p* Value
**Gram stain, n (%)**			
Gram-positive bacteria	57 (91.9)	9/9 (100)	1.000
Gram-negative bacteria	8 (8.1)	0 (0.0)	
**Pathogen, n (%)**			
MSSA	43 (69.4)	3/9 (33.3)	**0.038**
*Streptococcus* and *Enterococcus* spp.	5 (8.1)	3/9 (33.3)	
Enterobacterales	4 (6.5)	0 (0.0)	
Coagulase-negative *Staphylococci*	5 (8.1)	0 (0.0)	
MRSA	1 (1.6)	1/9 (11.1)	
Anaerobic bacteria	3 (4.8)	2/9 (22.2)	
Pseudomonas aeruginosa	1 (1.6)	0 (0.0)	
**Polymicrobial**	0 (0.0)	3/9 (33.3)	**0.003**
**Diagnostic sensitivity, n (%)**			
Blood culture	29 (46.8)	5/9 (55.6)	0.729
Intraoperative specimen	59 (95.2)	6/9 (66.7)	**0.024**
CT-guided biopsy of psoas abscess	11/19 (57.9)	0/2 (0.0)	0.214

SIA: spinal intradural abscess; SEA: spinal epidural abscess; MMSA: methicillin-sensitive *Staphylococcus aureus*; MRSA: methicillin-resistant *Staphylococcus aureus*; CT: computer tomography. Values in bold are significant results (*p* < 0.05), as indicated in the methods.

**Table 3:** Antibiotic management and surgical features of spinal intra- or epidural abscess.

Variables	SEA (n = 66)	SIA (n = 12)	*p* Value
**Type of antibiotic strategy, n (%)**			
EAT	51 (77.3)	10 (83.3)	1.000
TAT	15 (22.7)	2 (16.7)	
**Antibiotic treatment, mean ± SD**			
Time passed to TAT, D	4 ± 3	6 ± 3	0.366
Duration of intravenous antibiotics, W	3 ± 2	7 ± 4	**<0.001**
Total duration of antibiotics, W	7 ± 3	12 ± 10	**0.022**
CrP level, mg/L	27 ± 31	49 ± 61	0.359
Leukocyte count/L	7 ± 3	9 ± 4	0.119
**Surgical characteristics**			
Incidental dural tears, n (%)	7 (10.6)	--	--
Time passed to surgery, mean ± SD, D	2 ± 3	2 ± 2	0.864
Postoperative MS score, mean ± SD	90 ± 20	35 ± 44	**<0.001**
Paravertebral psoas abscess n (%)	33 (50.0)	2 (16.7)	0.056
Pleural empyema, n (%)	9 (13.6)	7 (58.3)	**0.002**
**Number of decompressed levels**			
I	32 (48.5)	6 (50.0)	0.763
II	21 (31.8)	4 (33.3)	
III	6 (9.1)	2 (16.7)	
IV	5 (7.6)	0 (0.0)	
V	2 (3.0)	0 (0.0)	
**Number of required surgeries, n (%)**			
I	57 (86.4)	8 (66.7)	**0.009**
II	8 (12.1)	1 (8.3)	
III or more	1 (1.5)	3 (25.0)	

SIA: spinal intradural abscess; SEA: spinal epidural abscess; TAT: targeted antibiotic treatment, EAT: empirical antibiotic treatment; SD: standard deviation; D: days; W: weeks; CrP: C-reactive protein; MS: motor score of the American Spinal Injury Association grading system. Values in bold are significant results (*p* < 0.05), as indicated in the methods.

**Table 4:** Complications and hospitalization rates between spinal intra- or epidural abscess patients.

Variables	SEA (n = 66)	SIA (n = 12)	*p* Value
**Complications, n (%)**			
Sepsis	12 (18.2)	9 (75.0)	**<0.001**
Septic embolism	4/48 (8.3)	4 (33.3)	**0.043**
Sign of meningism	12/65 (18.5)	12 (100)	**<0.001**
Infective endocarditis	1/52 (1.9)	0 (0.0)	1.000
Ventriculitis or cerebral abscess	2 (3.0)	5 (41.7)	**<0.001**
Deep wound infection	9 (13.6)	2 (16.7)	0.675
Pneumonia	9 (13.6)	7 (58.3)	**0.002**
Urinary tract infection	10 (15.2)	4 (33.3)	0.212
Re-Surgery	9 (13.6)	3 (25.0)	0.383
Relapse	4/46 (8.7)	2/8 (25.0)	0.213
Disease-related mortality	1 (1.5)	4 (33.3)	**0.002**
**Hospitalization, mean ± SD, D**			
Hospital stay	26 ± 20	58 ± 36	**<0.001**
ICU stay	4 ± 8	14 ± 18	**0.002**

SIA: spinal intradural abscess; SEA: spinal epidural abscess; SD: standard deviation; D: days; ICU: intensive care unit. Values in bold are significant results (*p* < 0.05), as indicated in the methods.

## Data Availability

The original contributions presented in this study are included in the article. Further inquiries can be directed to the corresponding author.
